# The Citizen as Contributor—Letters to the Editor in the Austrian Tabloid Paper *Kronen Zeitung* (2008–2017)

**DOI:** 10.1080/1461670X.2019.1702476

**Published:** 2020-06-01

**Authors:** Lore Hayek, Manuel Mayrl, Uta Russmann

**Affiliations:** a Department of Political Science, University of Innsbruck, Innsbruck, Austria; b Department of Communication, FHWien der WKW University of Applied Sciences for Management & Communication, Wien, Austria

**Keywords:** Letters to the editor, public discourse, tabloid newspapers, public opinion, elections, content analysis

## Abstract

This paper addresses the subject of letters to the editor as one of the longest standing forums for public discussion and debate by ordinary citizens. To show how the voice of ordinary citizens is presented in letters to the editor during national election campaigns over a period of ten years (2008, 2013 & 2017), we are focusing on the Austrian *Kronen Zeitung:* A newspaper with an exceptionally high market share of up to 40% during the examination period, a heavy focus on the letters section with three pages per day, and a self-declared willingness to take a stance, especially during election periods. Based on a quantitative content analysis of 530 letters to the editor and 525 articles in the politics section as well as survey data from the Austrian national election study on the political positions of the *Kronen Zeitung’s* readers, we find that letters to the editor in the *Kronen Zeitung* do not reflect, but complement the articles in the politics section. The tone of the letters is more negative than that of news articles, but the letters closely reflect the readers’ political positions, therefore offering identification with the paper.

## Introduction

Letters to the editor is one of the longest standing forums for public discussion and debate by ordinary citizens (Gregory and Hutchins 2004; Hynds 1994; Wahl-Jorgensen 2002a). Letters to the editor reflect a broad spectrum of public opinion as they allow the public to introduce topics, thereby revealing citizens’ thoughts and attitudes and capturing various opinions and arguments (Grey and Brown 1970; Wahl-Jorgensen 2002a). “One of the functions of the letters to the editor in a democratic society is that of catharsis. A letter column gives the irate, the antagonist, the displeased, a chance to speak out and to be heard” (Grey and Brown 1970, 454).

Even in times of online newspapers, which offer greater possibilities for citizens to express their opinion through online comments (see, for example, McCluskey and Hmielowski 2012), the letters to the editor section in newspapers can be considered “a key institution of the public sphere” (Wahl-Jorgensen 2002a, 69; see also Gregory and Hutchins 2004; Richardson and Franklin 2004). Letters to the editor are highly valued by their readership (Raeymaeckers 2005), indeed, research shows that they are among the best-read items of a newspaper (Hynds 1994), and usually read by far more people than online commentaries. Newspapers themselves highlight the democratic potential of letters to the editor, as these foster participation of ordinary citizens in public discourse (Richardson 2008; Wahl-Jorgensen 2001, 2002b) and “assist readers for making decisions and taking action on issues” (Hynds 1994, 573). This closely relates to the idea of deliberative democracy. Stressing the gatekeeping function of professional journalists for letters to the editor sections and the letters’ predominantly reactive nature, Nip (2006) and other scholars commonly locate them in the field of traditional journalism. However, as Lewis, Kaufhold, and Lasorsa (2010) point out, editorial oversight and gatekeeping also take place in the digital sphere, i.e., on online newspaper sites. Additionally, the subject of our study, the Austrian *Kronen Zeitung*, is well known for its emphasis on readers’ letters, referring to their readers even as co-creators of the newspaper. Thus, we also situate our study in the field of participatory journalism, which is commonly described as “the process in which citizens contribute to professional journalists’ news production” (Abbott 2017, 283) more or less independently.

This study examines how the voice of ordinary citizens is represented in letters to the editor in Austria’s largest newspaper, the tabloid paper *Kronen Zeitung*, during national election campaigns over a period of ten years. The *Kronen Zeitung* reaches about 2.2 million readers daily (29% of the Austrian population, Media-Analyse 2017), one of the highest market shares in readership in any democratic country (Hallin and Mancini 2004). More than any other newspaper in Austria, the *Kronen Zeitung* focuses on the letters section and shows a self-declared willingness to take a stance especially during election periods. To show how the voice of ordinary citizens is presented in letters to the editor in tabloid newspapers during national election campaigns over a period of ten years, we analyzed the content of 530 letters to the editor published in the Austrian *Kronen Zeitung* during the last three Austrian national election campaigns, 2008, 2013 and 2017, and compared them to 525 articles in the politics section of the *Kronen Zeitung*, which is where the majority of coverage on national elections can be found (Lengauer 2007). The study focuses on election campaigns as this allows us to analyze whether letters to the editor reflect a plurality of voices, or are no more than “hazy reflections of public opinion” (Grey and Brown 1970, 450). To illustrate the reflection of the readership’s views on the national elections in the politics and the letters to the editor sections of the *Kronen Zeitung*, we drew on election survey data from the Austrian National Election Studies (AUTNES) in 2008, 2013 and 2017 (Kritzinger et al. 2014, 2017, 2018), which revealed the most important issues for the regular *Kronen Zeitung* readers.

The paper begins with a discussion of the letters to the editor section as a forum for public deliberation, followed by a discussion of the limited number of studies on this subject, from which we draw our research questions and hypotheses. We will also describe the specific setting of this study by putting a spotlight on the *Kronen Zeitung.* Before presenting the results, we will explain the design of the study. The final section includes a discussion on whether the letters to the editor section in the *Kronen Zeitung* does indeed serve as a forum for ordinary citizens to present their opinions and arguments, as emphasized by their long-time editor Hans Dichand, and therefore reflects a broad spectrum of public opinion.

## Literature Review: Letters to the Editor

### A Forum for Public Deliberation and Characteristics of Letter Writers

Following the notion of deliberative democracy (Habermas 1992),

democratic politics involves *public deliberation focused on the common good*, requires some form of *manifest equality* among citizens, and *shapes the identity and interests* of citizens in ways that contribute to the formation of a public conception of the common good. (Cohen 1989, 19)

Diversity of the participants is an essential aspect of deliberation. A plurality of voices is to be heard, upon which citizens base their informed decisions and make public judgments about common preferences (Habermas 1984). Letters to the editor are a unique form of voluntary political participation (Cooper, Knotts, and Haspel 2009) that has existed almost as long as mass newspapers themselves. Equal citizens commit themselves to strive toward the common good by resolving their diverging stances through dialog and reason (Cohen 1997).

By engaging in deliberation, citizens acknowledge the possibility that they may change their preferences. The preferences that they assert now may not be the preferences they find they wish to express later. The very nature of the deliberative process of justification sends a signal that its participants are willing to enter into dialogue in which the reasons given, and the reasons responded to, have the capacity to change minds. (Gutmann and Thompson 2004, 20)

Thereby, the most just decisions can be reached and “bring about a truly informed ‘consent of the governed’” (Wahl-Jorgensen 2001, 305). Public deliberation is an indispensable precondition for the legitimacy of democratic institutions (Benhabib 1996).

The role of the media in public deliberation is predominantly one of information provision for citizens. For the most part, media coverage reflects and reproduces social inequalities, e.g., by privileging the opinions of people in power and disregarding those of ordinary citizens. However, in the past, newspapers also acted as “enablers of participation” (Nielsen 2010, 21) and editors incorporated a normative-economic claim to serve the community by providing “a ‘wide open public forum’, central to the practice of democracy”, while simultaneously serving the newspapers’ financial interests (Wahl-Jorgensen 2002b, 130). In their letters to the editor section, the newspapers aim to give ordinary citizens a voice, enter into a discourse with other citizens and/or political elites as well as enable them to “contribute to public debate in a spontaneous and uncoordinated way” (Richardson and Franklin 2004, 476; see also Nielsen 2010). By giving ordinary citizens a voice in letters to the editor, the media also gains knowledge about the public agenda and issues that are of interest to ordinary citizens.

Indeed, while this form of participation is open to anyone, critique has been raised regarding whether letter writers, as well as the content of the letters, represent public opinion. Letter writers are described as an “articulate minority” (Gallup 1958 as cited in Grey and Brown 1970, 454), as they are in number rather small, but tend to write repetitively (Buell 1975; Converse, Clausen, and Miller 1965). In the case of the *Kronen Zeitung*, some regular letter writers became small-scale “celebrities” and identified themselves outside of the letters’ pages (Borgers 2002). Studies show that letter writers are mainly men, well-educated, older and leaning conservative (Buell 1975; Cooper, Knotts, and Haspel 2009). Thus, they especially represent those people who are more willing to participate politically, but who are far from being part of the political elite (Buell 1975). While there is literature which suggests that some aspects of letters to the editor are “canned” letters (Reader 2005), whereby interest groups or political parties provide messages or even full letters for their supporters to send to newspapers (Richardson and Franklin 2004), there is no evidence that letter writers are not in fact genuine people. Furthermore, editors perform an editorial gatekeeping function as they recognize circular letters and prefer ones that are “written in as private individuals, expressing their thoughts on the issue in their own words” (Wahl-Jorgensen 2001, 312) instead.

However, the consistency of deliberative ideals and ordinary citizens’ motivation to write letters to the editors is disputable. Typically, researchers assume that letters to the editor perform a safety valve function for letter writers, a possibility to vent their displeasure (Buell 1975; Foster and Friedrich 1937). Drawing on the case of the Kent State shootings, Lander (1972) suggested that letters to the editor are better understood as potentially dangerous pressure valves as they shape the political environment in a community and might even encourage violence. Nevertheless, although letters to the editor may not perfectly meet the normative standards of public deliberation, they provide an important possibility for ordinary citizens to contribute to and shape the public discourse “side by side with formal representatives in a shared and in principle equal space of representation” (Nielsen 2010, 33). Furthermore, letters to the editor can influence political responsiveness as they provide politicians with an opportunity to understand public opinion (Herbst 1998). Hence, and following current empirical research on letters to the editor (see, for example, Gregory and Hutchins 2004; Richardson and Franklin 2004; Wahl-Jorgensen 2001, 2002a, 2002b), this study argues that “letters to the editor are an indispensable forum for public debate” (Richardson and Franklin 2004, 459; see also Richardson 2008).

Following this theoretical discussion on letters to the editor reflecting the voice of ordinary citizens, we go beyond previous research in the field by comparing the findings on the letters with election survey data of 2008, 2013 and 2017, identifying the most important issues for regular *Kronen Zeitung* readers. Hence, the first hypothesis predicts:

Hypothesis 1: The letters to the editor reflect the position of the Kronen Zeitung’s readership on policy issues.

### Editorial Selection Strategies

Albeit deliberative ideals, editors often select, frame and sometimes even edit letters not only according to their newsworthiness, but “in accordance with the identity of the newspaper, the (often only perceived) preferences of the readership, and other more mundane requirements of space and balance” (Richardson and Franklin 2004, 462). Hence, editors determine whose voices are heard based on routine selection practices (Bischofberger 2007; Gregory and Hutchins 2004; Wahl-Jorgensen 2001, 2002a, 2002b) and therefore critique has been made that editorial practices systematically construct and shape public opinion (i.e., public discourse) (e.g., Nielsen 2010; Torres da Silva 2012) and “challenge the ideals of rationality and deliberation” (Wahl-Jorgensen 2001, 303). Consequently, authors like Grey and Brown (1970, 454) assume that “the letters columns clearly are not representative of public opinion”.

Based on in-depth interviews with editors at San Francisco Bay Area newspapers, Wahl-Jorgensen (2002a) distinguishes four selection strategies: rules for relevance, brevity, entertainment and authority. The rule of relevance captures whether a letter to the editor responds to a current issue or newsworthy events on the agenda, the rule of brevity highlights that editors prefer short and punchy letters, the rule of entertainment refers to the spectacular tone of letters, i.e., letters which are provocative and “emotionally charged stories of individuals” (Wahl-Jorgensen 2001, 303) have a greater chance of being published, and the rule of authority suggests that editors prefer well-written letters (no grammar mistakes or unconventional styles of writing). These rules privilege certain forms of expression and, hence, editors “standardize the public debate of letters to the editor” (Wahl-Jorgensen 2002a, 70).

In her analysis of editorial practices in six Flemish newspapers, Raeymaeckers (2005) showed that editors of both tabloid as well as quality papers judge on the rules of relevance and brevity. The rule of entertainment applies mainly to letters to the editor in tabloid papers, “they provide a sounding board for topics that appeal to the ordinary reader” (Raeymaeckers 2005, 218), while the rule of authority applies more to letters in quality papers. Similar to the *Kronen Zeitung* (Borgers 2002), editors of Flemish tabloid papers try to include letters to the editor in order to give every citizen a voice—“they regard their editing as a sign of respect for the opinion of the less able letter writer” (Raeymaeckers 2005, 218).

### Case Study: The Kronen Zeitung

A comparatively high newspaper circulation characterizes the Austrian media landscape. Newspapers are the main source of information in Austria, and the tabloid paper *Kronen Zeitung* is Austria’s largest newspaper with one of the highest market shares in any democratic country. In the early 2000s, almost 50% of Austrian citizens read the *Kronen Zeitung* every day, and although recent years have seen a decline in readership, throughout the examination period of this study, it remained Austria’s largest newspaper by a large margin. In 2009, it reached 2.8 million readers daily (40.4%) and in 2017, it still reached 2.2 million readers (29.2%). The Sunday edition regularly exceeds these numbers even further (Media-Analyse 2009, 2017).

The *Kronen Zeitung*’s stance is rooted in its long-time editor Hans Dichand, who re-founded the newspaper after the Second World War in 1959 and served as editor-in chief until his death in 2010. His son Christoph Dichand succeeded him and continues to serve as editor today. Hans Dichand, who frequently and frankly spoke and wrote about his views on his newspaper’s role (e.g., Dichand 1977, 1996), saw himself as one of the figureheads of the fourth estate, ascribing certain powers of campaigning and persuasion to his newspaper. In his autobiography “Im Vorhof der Macht” (“In the antechamber of power”, Dichand 1996), for example, he claims co-responsibility for the success of the Konrad-Lorenz petition for animal protection (1984) and the referendum for Austria’s accession to the European Union (1994). Apart from taking a stance on different kinds of policy issues, the *Kronen Zeitung* is considered especially influential during election campaigns thanks to its numerous and diverse readers, many of whom read the *Kronen Zeitung* exclusively (Schoen 2010).

The letters to the editor play a very important role in defining the *Kronen Zeitung*’s power. Hans Dichand described his newspaper as the “megaphone for the vox populi” (“Megaphon für die Vox Populi”) (Borgers 2002) and being committed to free speech, he named the letters to the editor section “The free word” (“Das freie Wort”), as well as referring to his readers as “co-creators” of the newspaper. He once noted that “We treat our letters to the editor with particular care. This clearly shows that all our ‘Krone’ readers have a say in by far the largest newspaper in our country” (“Unsere Leserbriefe werden natürlich besonders pfleglich behandelt. Wir zeigen damit deutlich, dass wir alle, die unsere ‘Krone’ lesen, an dieser weitaus größten Zeitung unseres Landes mitgestalten lassen” (Der Standard 2009)). The letters remained in Dichand’s own domain up until his last years at work (Bischofberger 2007). Seven days a week, the letters to the editor section dedicates 2–3 pages of the newspaper to giving ordinary citizen a voice in the news. Over the years, this section has attracted a number of regular contributors, stating that they write several letters a week (Borgers 2002).

During election campaigns, the letters to the editor play a significant role in the *Kronen Zeitung*’s campaigning efforts—two examples will illustrate that function. In the 1986 presidential race, the *Kronen Zeitung* openly supported the ÖVP candidate Kurt Waldheim, whose membership in the SA (Sturmabteilung) and military activities during World War 2 became known to the public during the election campaign. Within the *Kronen Zeitung*’s massive pro-Waldheim campaign (Mitten 1992), some letters to the editor reflected explicit antisemitic sentiments. Dichand defended these by saying that “in our newspaper, readers may write what they really think” (Dichand 1996, 442). In the spring of 2008, social democratic chancellor Alfred Gusenbauer and party leader Werner Faymann chose a letter to the *Kronen Zeitung* as a medium to announce a shift in their stance on the ratification of EU treaties, calling for popular referendums on all future treaties and thereby adopting the long-time position of the newspaper (Luther 2009). This letter was the final trigger for calling early elections in the autumn, a campaign during which the *Kronen Zeitung* openly supported Faymann, including a double-paged “Who would animals vote for?” feature (Seethaler and Melischek 2013).

While letters to the editor will usually be in line with the newspaper’s overall agenda (Gregory and Hutchins 2004; Nielsen 2010), the history of the *Kronen Zeitung,* as presented in this section, suggests that the letters are not necessarily a mirror-like reflection of the newspaper’s daily coverage, but rather an opportunity to present additional views or issues, as shown also by Richardson and Franklin (2004). Our second hypothesis therefore reads:

Hypothesis 2: The issues touched upon in the letters to the editor complement the issue agenda of the Kronen Zeitung.

### Content of Letters to the Editor

Research shows that letters to the editor are closely related to the ongoing debate in the newspaper (Gregory and Hutchins 2004; Nielsen 2010). By analyzing the focus of letters to the editor in a regional newspaper in Australia, Gregory and Hutchins (2004) showed that local and state-based issues (65%) dominated these. Only a quarter of the analyzed letters focused on national and international issues. Coding for specific policy issues in North Carolina newspapers by Cooper and colleagues (2009) revealed that the issue addressed most often in letters to the editor was state and local administration, followed by defense, government operations, law, crime, and family issues, as well as education (top 5 issues). Overall, half of the letters focused on local politics (34%) and state politics (17%), and the other half (49%) addressed national politics. Richardson (2008) reveals similar results for the UK; letters to the editor in the *Sun*, *The Times* and the *Evening Post* “reflect the wider editorial content of the newspaper” (Richardson 2008, 61). However, letters to the editor were much more diverse regarding the topics in *The Times* and the *Evening Post*. In the *Sun,* three topics, crime/law & order, celebrity and race/immigration, represented almost half of the readers’ letters.

In terms of the tone of letters to the editor, studies reveal that local issues evoke more professional, respectful, objective, and personal tones and they are less polarized than nationally and internationally focused letters, which are more associated with angry, superior, and posing tones (Perrin and Vaisey 2008). Overall, however, research shows that letters to the editor tend to be more negative and cynical than positive (Cooper, Knotts, and Haspel 2009; Grey and Brown 1970; Lander 1972; Nielsen 2010; Richardson and Franklin 2004). Recent studies also reveal a more negative tone of opinions in letters to the editor than in online posts (McCluskey and Hmielowski 2012). Letters to the editor are a possibility for the public “to declare grievances and tell stories of a much more personal nature” (Richardson and Franklin 2004, 460). Or as Lander (1972, 142) noted, they provide the middle-aged and middle class conservative white Americans “a catharsis to blow off steam in an unreasoned and emotional way”. Grievance, criticism, disagreement and personal views spark the public debate (e.g., Nielsen 2010). On the contrary, newspapers in Austria follow primary journalistic norms for good quality such as objectivity and balance (see for example Kaltenbrunner, Lugschitz, and Gerard-Wenzel 2018). We assume that the selection of letters to the editor, just as in other newspaper sections, reflects deliberate choices made by the editor regarding issues, tone and relevance for the readership.

In line with this previous research (Cooper, Knotts, and Haspel 2009; Grey and Brown 1970; Lander 1972; Nielsen 2010; Richardson and Franklin 2004), we hypothesize that letters to the editor will tend to be more negative than articles in the politics section.

Hypothesis 3: Letters to the editor are more negative than articles in the politics section.

## Research Setting

### Content Analysis of Letters to the Editor and Articles

The data for this study was collected through a quantitative content analysis of newspaper articles in the politics section and letters to the editor in the *Kronen Zeitung*. We examined the last four weeks before the 2008, 2013, and 2017 Austrian national elections, the so-called hot phase of the campaign (Jakubowski 1998) (30 August to 27 September 2008; 1 September to 28 September 2013; 17 September to 14 October 2017). Reports and letters were selected if they contained the word or word stem “wahl” (election).

Our overall dataset includes 2273 articles in the *Kronen Zeitung*. For the analysis, we used 525 articles in the politics section and 530 letters to the editor. The politics section has been included due to its focus on national elections (Lengauer 2007). Together, these two forms comprise 1055 articles, almost half the election-related pieces published in the *Kronen Zeitung* during the examination period (Table 1).

**Table 1.** Number of articles in newspaper sections in the Kronen Zeitung, 2008–2017.

Section	2008	2013	2017	Total
Local news	97	640	88	825
Letters to the editor	337	99	94	530
Politics	270	159	96	525
Other sections	13	63	114	190
Business	29	44	4	77
Culture and communication	11	29	5	45
Title page	23	13	6	42
Sports	1	27	0	28
Supplements	3	0	8	11
Total	784	1074	415	2273

Comparing the number of letters to the editor and articles in the politics section across the three analyzed elections, results show that the number of letters to the editor has decreased over time (Table 1). The decrease of letters to the editor from 2008 to 2013 might be due to the editorial change in 2010 when Hans Dichand’s son became editor of the Kronen Zeitung, because the distribution of letters stabilizes again in the following years.

In this study, we focus on two characteristics of the articles analyzed, the article’s *main issue* and its *tone*. The main issue is the first content-related aspect, which is discussed in the broadest sense and is the answer to the question, “What is the story about?”. The issues were classified into 38 issue categories of which three are polity issues^1^ that deal with structural circumstances in the political process, six are political issues^2^ that include relationships between political actors and issues related to the election campaign, and 29 are policy issues.^3^

We measured the tone of an article on a three-point scale from −1 to +1, where articles that were coded −1 only focused on negative aspects such as unsolved problems, threats and conflicts related to the respective issues, and articles that were coded +1 focused on positive solutions, achievements and progress. Ambivalent and neutral articles were coded 0. When defining the respective issue categories, the project’s aim was to define neutral issues in order for the tone to cover only opinions from writers and not the topic. For instance, the issues of EU and taxes are neutral, but can be framed as either negative or positive by the tone applied. Following Lengauer and colleagues (2012), tone is defined as the overall tone of a news report, abstracted from specific topics and actors. We are aware that this operationalization does not fully take into account that some issues have, by nature, more negative connotations (e.g., crime)—however, it serves our purpose of comparing two fundamentally different forms of journalistic writing.

The coding processes for the 2008 and 2013 data took place within AUTNES—the Austrian National Election Study, and for the 2017 data in a project at the University of Innsbruck. Six to ten coders conducted the coding. Intercoder reliability scores were calculated using Holsti’s co-efficient of reliability (for 2008 and 2013) and Krippendorff’s Alpha (for 2013 and 2017). Overall, intercoder percentage agreement for each of the items falls within the acceptable range. The categories of the variable “main issue” used in this analysis were aggregated from (up to) three levels of subcategories applied by the different coder teams in different years. The coefficients for main issue are 0.75 (Holsti) in 2008, 0.76 (Holsti) and 0.78 (Krippendorff’s α) in 2013 and 0.74 (Krippendorff’s α) in 2017. Specific reliability statistics for the 2008 and 2013 datasets are stored in the AUTNES archive.^4^ Reliability statistics for the 2017 dataset are available from the authors.

### (Post-)election Survey Data

To compare the coverage of the *Kronen Zeitung* with the opinion of its regular readership we drew on election survey data from the Austrian National Election Studies (AUTNES) in 2008, 2013 and 2017 (Kritzinger et al. 2014, 2017, 2018).

We operationalized regular readers as survey respondents claiming to read the *Kronen Zeitung* at least two days a week (*N* = 1693, see Table 2). For the analysis, we included another four survey items^5^: Firstly, we included the question regarding readers’ perception of the most important issue. This allowed us to compare the salience of each issue between the *Kronen Zeitung* and its readership. We operationalized issue salience among readers as a relative number of respondents naming a certain issue as the most important one. Secondly, to assess policy stances among the newspaper’s readership, we identified issues that were both salient among readers and represented by comparable survey questions in the datasets. Three issues met our criteria and hence the following three survey items were included: attitudes towards European integration, migration and crime prevention. To increase comparability with tone of articles, we recoded the five- and ten-point Likert scale survey questions to a three-point scale from −1 to +1. Table 2 lists the number of respondents answering each survey item by year.

**Table 2.** Survey items and number of respondents from Austrian National Election Study (post-) post-election surveys.

Survey Item	2008	2013	2017	Total
Regular Readership	476	584	633	1693
Most important issue	438	103	211	752
European Integration	458	564	453	1475
Migration	470	574	464	1508
Crime prevention	474	574	–	1048

## Results

### The Kronen Zeitung and the Voice of Ordinary Citizens

To test our first hypothesis, we examined whether the letters section of the newspaper reflects the opinions of the newspaper’s regular readers, compared to the politics section. We tested this hypothesis with the three most salient policy issues in the readers’ letters: migration and integration, EU, and crime prevention (see also Figure 2). We compared the salience of issues in the two newspaper sections with the percentage of readers naming them the “most important issues” (both percentages), and the tone of the newspaper items on these issues with the policy stance of the readers (both ranging from −1 to +1).

**Figure 1.**
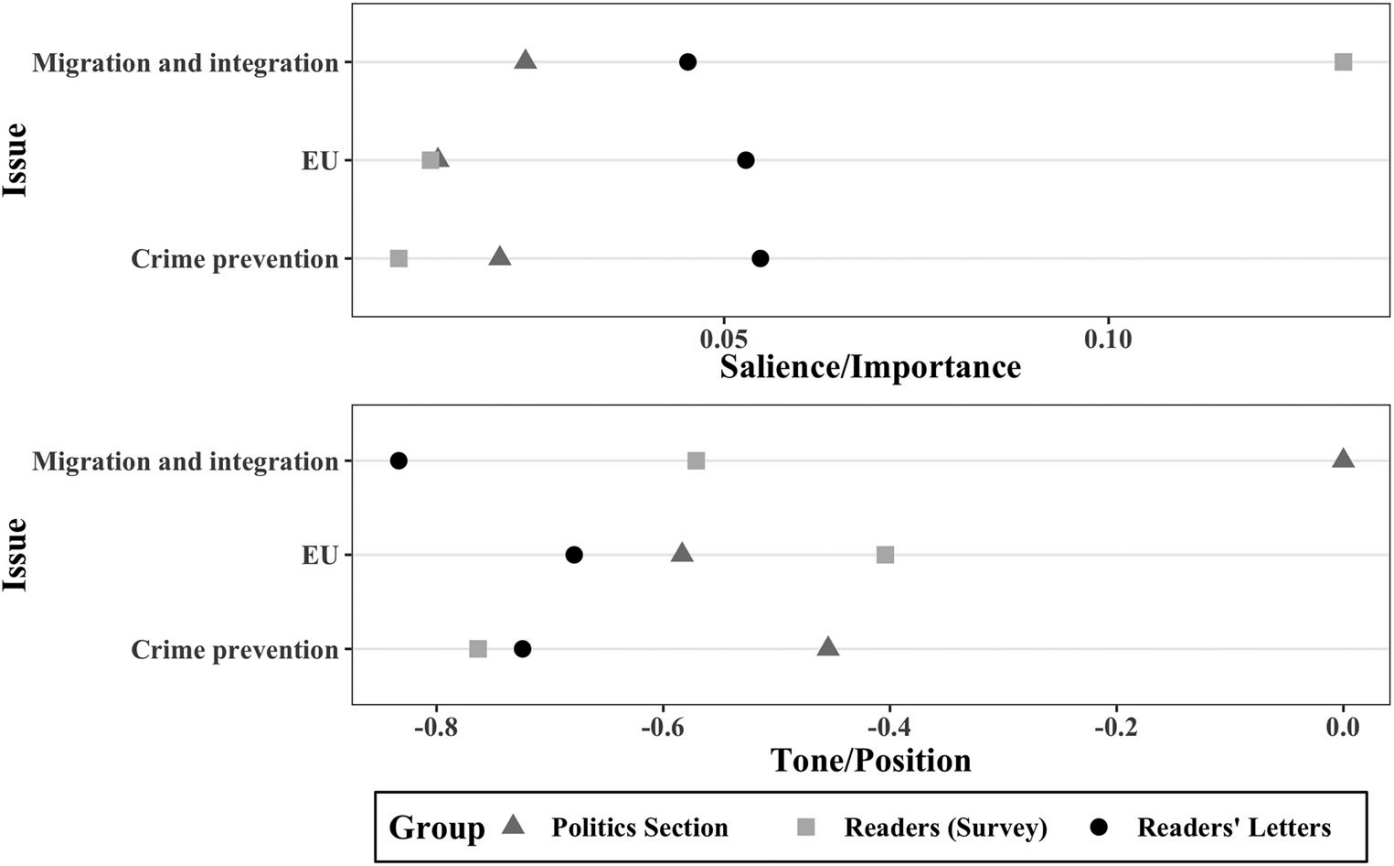
Comparison of tone and salience of issues among newspaper sections and reader’s preferences (2008–2017).

Figure 1 shows tone and salience of the three issues between the *Kronen Zeitung*, in both the politics and the letters section, and its readers’ preferences. All three issues are covered in similar frequency in the letters section, the same applies to the politics section. While the latter replicates readers’ issue salience in two of the three issues (EU and crime prevention) rather closely, the letters section does not reflect readers’ issue salience at all. For instance, the survey data shows that migration and integration is far more important to readers than how it is covered in the *Kronen Zeitung*.

We can confirm the initial impression that salience of policy issues differs between the three groups (Chi^2 ^= 12.551, *p* = 0.002). Pairwise comparison using Wilcoxon shows significant differences between readers’ letters and both the politics section (*p* = 0.005) and the readership’s preferences (*p* = 0.003).

On the other hand, Figure 1 suggests that the tone in the letters section better reflects readers’ policy positions in two of the three issues (crime prevention as well as migration and integration) compared to the politics section. Overall, the Kronen Zeitung’s politics section provides a more balanced coverage of the three issues than the letters section, i.e., less negative.

However, a Kruskal-Wallis rank sum test reveals that means of tone only differ significantly within the issue of migration and integration (*p* = 0.002) (Table 3). Pairwise comparisons using Wilcoxon reveals that this finding stems from a considerable difference between readers’ policy stance on migration and integration and the tone of the coverage of these issues in the *Kronen Zeitung*’s politics section. However, it is important to note that only two articles in the politics section, which focused on migration as its main topic, were coded as either positive or negative in tone.

**Table 3.** Pairwise comparison using Wilcoxon rank sum test (*p*-value adjustment method: Bonferroni).

		Migration	EU	Crime
Kruskal-Wallis	Chi-Squared	11.984	5.893	4.361
	df	2	2	2
	*p*-value	0.002	0.053	0.113
Wilcoxon		Reader’s policy stance (on issues above)		
Politics section		0.009	1.000	0.120
Reader’s letters		0.258	0.056	1.000

Hence, we cannot confirm the tendency depicted in Figure 1 that the politics section covers issues more balanced than the newspaper’s readership evaluates them. Additionally, readers’ preferences towards all three issues do not significantly differ from the readers’ letters. Thus, our results support hypothesis 1 that letters to the editor reflect the policy preferences of the *Kronen Zeitung*’s readership. In contrast, readers’ letters do not represent issue salience of the tabloids’ readers accordingly, i.e., issues are not touched upon as often in letters to the editor as their importance to regular readers would suggest. Consequently, high issue salience among readers does not necessarily lead to a higher proportion of letters to the editor addressing a certain issue.

### Content of Letters to the Editor and Articles in the Politics Section

**Figure 2.**
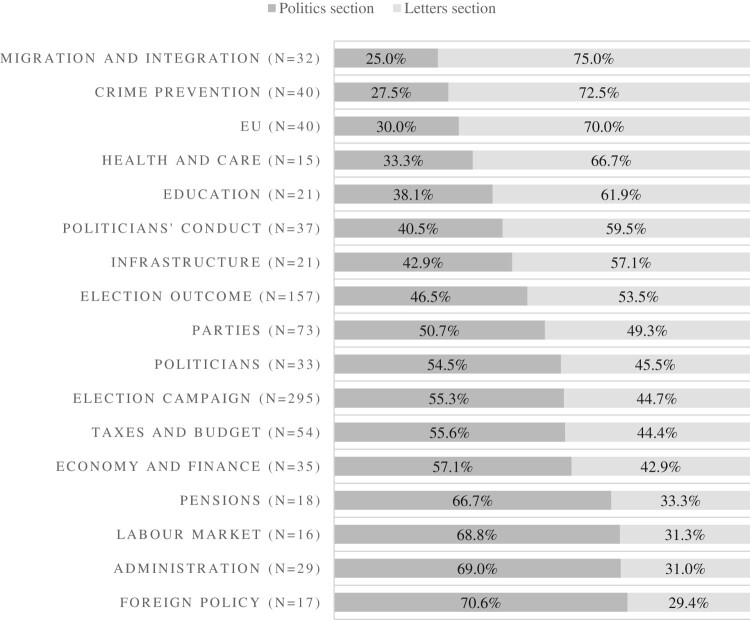
Issues in the politics and letters section of the *Kronen Zeitung*, 2008–2017 (All issues that appear in >15 articles are depicted).

Our second hypothesis predicted that the letters to the editor complement the news agenda of the *Kronen Zeitung*. Indeed, analysis of the content of letters to the editor and articles in the politics section based on their primary issue confirmed that the issues touched upon in the letters complement the issue agenda of the *Kronen Zeitung*. The results are displayed in Figure 2.

About one third of the contributions in the politics and letters sections dealt with substantial policy issues; almost two thirds covered politics issues on campaigns and candidates. The most broadly discussed issue was the electoral campaign itself (295 items), followed by the election outcome (157 items) and political parties (153 items). The most covered policy issues were taxes and budget (54 items), crime prevention (40 items) and the EU (40 items). However, the proportion of issues differs significantly between articles in the politics section and pieces in the letters section (Chi^2 ^= 62.58, *p* = 0.005). While issues such as the election outcome, characteristics of political parties and politicians as well as the election campaign in general were distributed equally between the two sections, it became very clear what letter-writers deeply care about. Migration and integration, crime prevention and the EU were the three most discussed policy issues in the letters to the editor. More than two thirds of the pieces on migration and integration, crime prevention and the EU in the analyzed material could be found on the letters pages. These findings are consistent with Richardson (2008), who found race/immigration, crime and celebrities to be most prominent in letters to the editor in the *Sun.* Issues such as the labor market, administration and foreign policy are predominantly covered in articles in the politics section (even though the Ns are rather small for these three issues) and of less interest to letter-writers.

### Tone of Letters to the Editor and Articles in the Politics Section

The third hypothesis predicted that letters to the editor were more negative than articles in the politics section. The tone of articles was measured on a −1 to +1 three-point scale.

Confirming the hypothesis, a T-test (*p* = 0.000) showed that the letters were significantly more negative than articles in the politics section: the mean value for tone is −0.244 for the politics articles, in comparison to −0.703 for letters to the editor. Not a single letter to the editor in the data was coded higher than 0 (neutral in tone).

**Figure 3.**
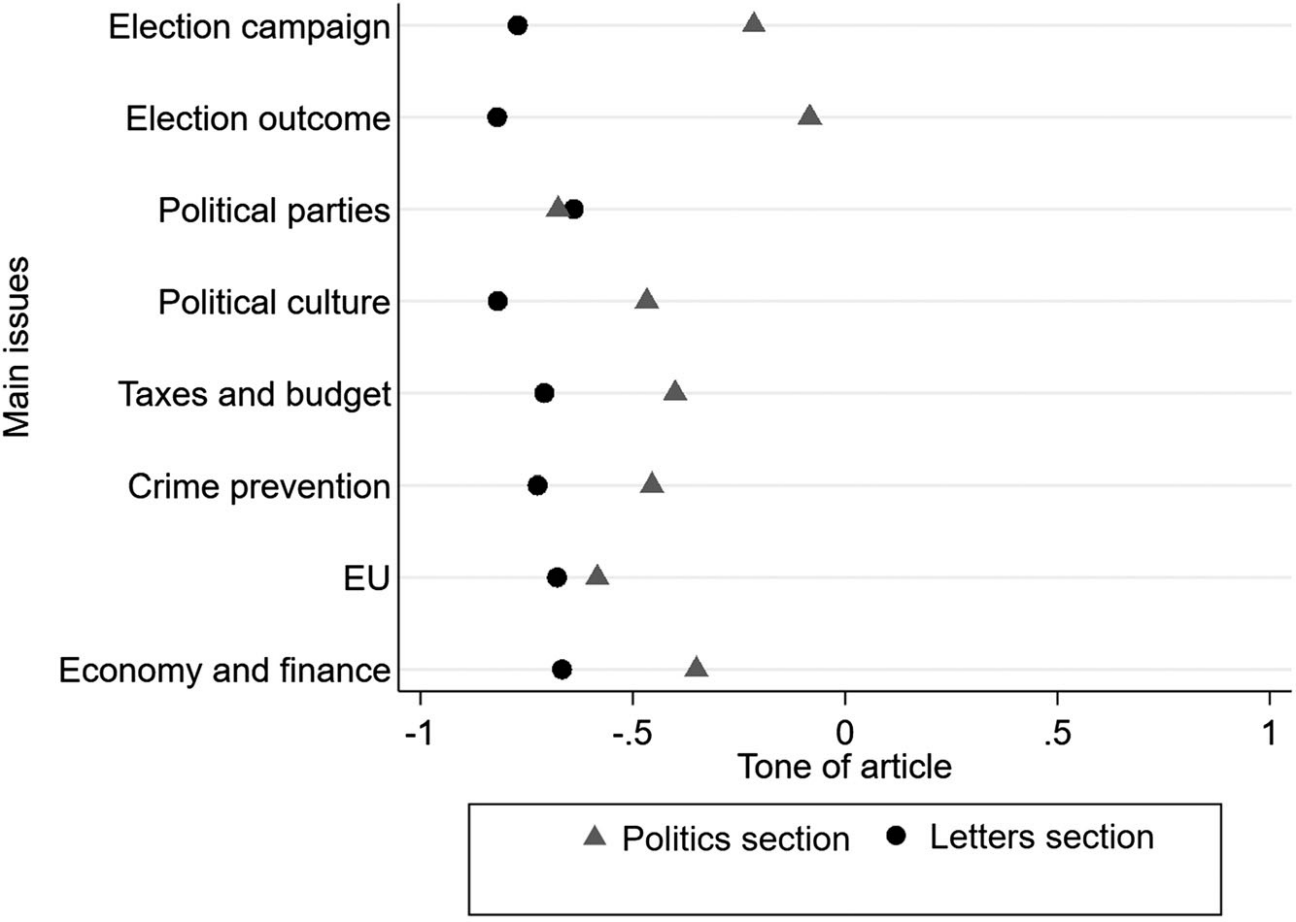
Tone for issues in the politics and letters sections that appear in >35 articles (2008–2017).

Figure 3 displays the differences in tone between the politics and letters sections for the most frequent main issues. Although there is a general negative tone on these issues in both politics and letters sections, readers are still more likely to be negative in rating the issues, with the exception of political parties. For the issue regarding the EU, both the letters and the articles are rather negative, and we found the largest differences in mean for the issues regarding the election campaign and election outcome. While the mean tone in the politics section is neutral on these issues, readers’ letters comment very negatively on them—this may be a result of a certain disenchantment with politics. On taxes and budget, crime prevention, economy and finance as well as political culture, none of the sections take a positive tone, but letter writers comment even more negatively.

## Discussion and Conclusions

This study makes an empirical contribution to the scarcely researched field of letters to the editor in tabloid newspapers and their relationship to public opinion. Given that the *Kronen Zeitung* strives to embody participatory journalism in a traditional print media outlet and to be in unison with its readership, it is particularly interesting that this aspect has not received more scholarly attention.

Consistent with H1, the policy preferences of the *Kronen Zeitung*’s regular readership are reflected in the readers’ letters. Drawing on 1693 respondents in post-election surveys in 2008, 2013 and 2017 and their policy preferences towards three issues (EU, crime prevention as well as migration and integration), our findings show that the tone of readers’ letters does not significantly deviate from the readers’ policy preferences. However, the amount of readers’ letters on these three issues misrepresents readers’ perceived importance of these issues. Firstly, letters covering the EU and crime prevention are overrepresented in comparison to their salience among readers. Secondly, more readers named migration and integration as the most important issue than suggested by its amount of coverage in the *Kronen Zeitung*. These results suggest that the *Kronen Zeitung*’s letters to the editor are not merely reflections of the newspaper’s editorial agenda, as suggested by Grey and Brown (1970). Instead, they seem to rather accurately reflect the policy stances of the newspaper’s readership and can therefore be perceived as an expression of participatory journalism. However, as the Kronen Zeitung’s readership is—although large in number—not representative of the Austrian population, we propose seeing its letters section as a *subforum* of public deliberation, not capable of forming a “consent of the governed” (Wahl-Jorgensen 2001, 305), but rather a consent among a newspaper’s readership.

Confirming H2, the letters to the editor are not a mirror-like reflection of the *Kronen Zeitung’s* politics agenda. Instead, they complement the tabloid paper’s agenda. The most frequently covered, and hence most salient, issues covered by letter writers were migration and integration, crime prevention and the EU. This (partly) confirms the results of Richardson (2008), who found that in the British *Sun* the issues of crime/law and order, race/immigration and celebrity represented almost half of the readers’ letters. These issues, migration and integration, crime prevention and the EU, were less prominent in the politics section of the *Kronen Zeitung*, which focused instead on topics such as taxes and budgets as well as economics and finances. Hence, the findings corroborate the *Kronen Zeitung’s* claims to be a provider of a forum for ordinary citizens to discuss and debate topics that are of importance to them. According to the post-election surveys, migration and integration was one of the most important issues for *Kronen Zeitung* readers over the past ten years. Letters on issues that are important to readers are often provocatively written and emotionally charged and a spectacular tone of letters is something editors foster (Wahl-Jorgensen 2001).

Consistent with H3, our findings show that the tone of letters to the editor is much more negative than in articles in the politics section. The policy issue regarding the EU stands out, with both sections being similarly negative—this can be tied to the *Kronen Zeitung*’s position towards the EU today, which is very skeptical overall, for instance, the *Kronen Zeitung* is generally calling for popular referendums on all EU treaties (Luther 2009). For many years, letters on this issue have been published under the headline “Leserbriefe zum EU-Theater” [“Letters on the EU drama”]. Overall, our findings are in line with several studies from other countries (Cooper, Knotts, and Haspel 2009; Grey and Brown 1970; Lander 1972; Nielsen 2010; Richardson and Franklin 2004). Grievance, criticism, disagreement and personal views appeal to the ordinary reader and spark the public debate (e.g., Nielsen 2010; Raeymaeckers 2005). Or, as Wahl-Jorgensen (2001, 310) puts it: “the letters to the editor section is justified not only as a democratic forum, but as a public relations tool and a revenuer booster for the newspaper”. Furthermore, our results are consistent with both the idea of writing a letter to the editor as a safety or pressure valve for ordinary citizens (Buell 1975), and of the rule of entertainment as an editorial selection strategy, especially in tabloid media (Raeymaeckers 2005; Wahl-Jorgensen 2002a). Congruent with the findings of our third hypothesis, the tone of the letters to the editor complements the politics section of the newspaper. Although the leaning on policy issues is the same, it does offer its writers and the newspaper the possibility to provide their readers with more unambiguous interpretations of political issues and events (Richardson and Franklin 2004).

To sum up our three hypotheses, as we assumed the policy positions of tabloid readers towards certain issues to be more negative than the editorial stance, the letters reflect these readers’ positions (H1), therefore readers’ letters complement the editorial issue agenda (H2) and are more negative in tone (H3).

Altogether, our findings add to as well as extend research in the field of letters to the editor. Specifically, future studies ought to investigate if letters reflect the general readership’s position on the issues covered as this opens a new direction on this string of research and gives greater insights into the voice of ordinary citizens in newspapers. For instance, a suitable further study would be a cross-country comparison with letters to the editor in large tabloids across Europe such as the German *Bild* and the British *Sun*.

As with any investigation, our study is not without limitations, but these also provide insights into future research avenues. Firstly, the decrease of letters to the editor from 2008 to 2013 supports previous research that emphasizes the strong role of the editor regarding the letters (Bischofberger 2007; Gregory and Hutchins 2004; Richardson and Franklin 2004; Wahl-Jorgensen 2001, 2002a, 2002b). Previous research has shown that by conducting interviews with editors, policies for running letters can be revealed. In our case this is somewhat restricted due to the death of Hans Dichand, the “founder” of “The free word”, in 2010—an obstacle that research over time sometimes must face. An interview with the succeeding and current editor, his son Christoph Dichand, can give some insight into current editorial practices, which can then be compared to interviews conducted with Hans Dichand, who referred to his readers as “co-creators” of the newspaper. Secondly, as shown in the study conducted by Nielsen (2010) on the Danish broadsheet *Politiken*, a comparison of printed letters to the editor with all letters received can shed further light on editorial practices of this very important section of the *Kronen Zeitung*. Such a comparison between received and printed letters to the editor will also reveal more about those who are writing them. Thirdly, letters to the editor these days have strong competition in the form of online forums. While some might argue that online comments as potential virtual agoras (see Muhlberger 2006) would be an interesting comparison with our data, we emphasize that they lack the element of editorial selection and embedment in an editorial context. While we know that letters to the editor is one of the most-read newspaper sections (Hynds 1994), we doubt that online comments receive the same attention as the news articles with which they are associated. Moreover, we treat letters to the editor as pieces of writing and the letter-writer as an author, while most online comments are short and often hastily written expressions of opinion.

The *Kronen Zeitung* is well aware that it is a powerful actor in shaping the political discourse in Austria, after all, the tabloid paper reaches almost a third of the Austrian population every day. However, following the notion of deliberative democracy, the newspaper also gives ordinary citizens a voice. The *Kronen Zeitung* does actually serve as a forum for ordinary citizens to present their opinions and arguments: Seven days a week, the letters to the editor section covers 2–3 pages of the newspaper. We empirically show in this paper that the letters to the editor are an important factor in the editorial construction of public opinion during election times (Schoen 2010). By allowing for a variety of issues in the letters, and for a tone that may be considered inappropriate elsewhere in the newspaper, the editors manage to include a cross-section of views and perceptions, helping them to satisfy all parts of their large and diverse readership. In that sense, the letters to the editor can be perceived as a strategic instrument to contribute to the size of the newspaper’s market share and therefore its economic success.

## Notes

1 Polity issues: Administration, electoral law, federalism.

2 Political issues: Coalition building, election results, election campaigns, parties, politicians’ personalities, politicians’ behavior.

3 Policy issues: Agriculture, budget and taxes, consumerism, crime prevention, culture, disaster relief, economy, environment, EU, families, financial market, foreign policy, health and care, higher education, human rights, infrastructure, kindergarten and schools, labor market, law and justice, media, migration and integration, military, pensions, religion, right-wing extremism, society, sports, terrorism, welfare state.

4 The archive is accessible at http://www.autnes.at.

5 Details on survey items are included in the appendix.

## Appendix

**Table A1.** Comparison of survey questions from AUTNES (post-)post surveys 2008, 2013 and 2017.

Survey Items	
Readership	
2008	Please tell me for each of the following daily newspapers, whether you read in them about Austrian politics daily, a few times a week, less often or never.
2013	Below you will find a list of different newspapers. On average, on how many days in a regular week do you read the print version of these papers?
2017	On average, on how many days in a regular week do you read these newspapers or websites?
Most important issue	
2008	What do you think is the most important political problem facing Austria today?
2013	Which issues are most important to you personally in the upcoming national parliamentary election on 29 September 2013?
2017	Which political issue in Austria is currently most important to you personally?
European Integration	
2008	Sometimes one hears that the European unification should be advanced further. Others say that it has already gone too far. What is your opinion? Please use the scale from 1 to 11, where 1 means that the European unification has already gone too far and 11 means that it should be driven further.
2013	Some say that European Integration has already gone too far, others say the European Integration should go further. What is your opinion, using a scale from 0 to 10, where 0 means that European Integration has already gone too far, and 10 means that European Integration should go further?
2017	Some say that the European unification has already gone too far, others say that the European unification should be pushed even further. What is your opinion on a scale from 0 to 10, where 0 means that the European unification has already gone too far, and 10 means that the European unification should be pushed even further?
Migration	
2008	One can have different opinions about different political topics. What about you: how true/accurate do you find the following statements on a scale from 1 to 11, where 1 means that it is very true and 11 that it is not true at all: Immigration to Austria should be significantly curbed.
2013	One can have different opinions on various political issues. We will now present to you a series of political statements. Please indicate to what extent you agree with the following statements: Immigration to Austria must be stopped.
2017	Do you completely agree, somewhat agree, partly agree, partly disagree, somewhat disagree or completely disagree with the following statements?: Immigration to Austria should only be possible in absolutely exceptional cases.
Crime prevention	
2008	One can have different opinions about different political topics. What about you: how true/accurate do you find the following statements on a scale from 1 to 11, where 1 means that it is very true and 11 that it is not true at all: Criminal offenders should be punished more severely.
2013	One can have different opinions on various political issues. We will now present to you a series of political statements. Please indicate to what extent you agree with the following statements: Criminals need to be punished severely.
2017	NA
